# Effects of 12 weeks resistance training on muscle quality and physical performance in normal-weight and obese older women

**DOI:** 10.3389/fphys.2026.1794580

**Published:** 2026-04-30

**Authors:** Nicolás Vidal-Seguel, Alexis Sepúlveda-Lara, Juan Carranza-Leiva, Carlos Márquez, Nolberto Huard, Jorge Sapunar, Luis A. Salazar, Estefanía Nova-Lamperti, Gabriel Nasri Marzuca-Nassr

**Affiliations:** 1 Programa de Doctorado en Ciencias Morfológicas, Facultad de Medicina, Universidad de La Frontera, Temuco, Chile; 2 Departamento de Ciencias Básicas, Facultad de Medicina, Universidad de La Frontera, Temuco, Chile; 3 Departamento de Procesos Terapéuticos, Universidad Católica de Temuco, Facultad de Ciencias de la Salud, Temuco, Chile; 4 Doctorado en Ciencias Mención Biología Celular y Molecular Aplicada, Facultad de Ciencias Agropecuarias y Medioambiente, Universidad de La Frontera, Temuco, Chile; 5 Departamento de Ciencias de la Rehabilitación, Facultad de Medicina, Universidad de La Frontera, Temuco, Chile; 6 Clínica de Medicina Física y Rehabilitación MEDIFIS, Unidad de Kinesiología, Temuco, Chile; 7 Departamento de Medicina Interna, Universidad de La Frontera, Facultad de Medicina, Temuco, Chile; 8 Núcleo de envejecimiento, vejez y calidad de vida, INTA, Universidad de Chile, Santiago, Chile; 9 Centro de Biología Molecular y Farmacogenética, Departamento de Ciencias Básicas, Universidad de La Frontera, Facultad de Medicina, Temuco, Chile; 10 Centro de Investigación e Innovación del Cáncer, Fundación Arturo López Pérez OECI Cancer Center, Santiago de Chile, Chile; 11 Departamento de Bioquímica Clínica e Inmunología, Laboratorio de Inmunología Molecular y Traslacional, Universidad de Concepción, Facultad de Farmacia, Concepción, Chile

**Keywords:** elderly, exercise, muscle quality, obesity, strength training

## Abstract

Ageing and obesity are major public health issues, both associated with reductions in muscle mass, strength, and physical performance. Muscle quality, which integrates the morphological and functional characteristics of skeletal muscle, is a key predictor of independence and mortality in older adults. Obesity is linked to additional structural and functional alterations in muscle during ageing, alongside a potential anabolic resistance. Although resistance training (RT) has been shown to improve muscle mass and quality in older adults, evidence in older women with obesity remains limited. This study aimed to compare the effects of a 12-week progressive, whole-body RT programme on muscle quality parameters and physical performance in women aged 60–79 years with obesity (68 ± 4.21 years; body mass index [BMI] = 33.01 ± 2.62 kg/m²; body fat percentage= 42.23% ± 2.98%; n = 16) versus age-matched normal-weight women (66 ± 4.31 years; BMI = 22.60 ± 1.36 kg/m²; body fat percentage= 33.11% ± 3.61%; n = 14). RT was performed three times per week for 12 weeks. Before and after the intervention, morphological indicators of muscle quality (quadriceps muscle thickness and echointensity) and physical performance were assessed. Both groups showed significant post-training improvements in quadriceps thickness (time effect, p < 0.001); maximal strength in leg extension, leg flexion, leg press, chest press, and elbow extension (p < 0.001), lower-limb power, and muscle quality index (p < 0.001), as well as physical performance (p < 0.001); In conclusion, although the increase in muscle thickness and the reduction in body fat percentage were only significant in the normal-weight group, 12 weeks of RT are effective in improving functional parameters of muscle quality and physical performance in normal-weight and obese older women. This supports RT as an effective strategy to optimise musculoskeletal health in this population.

## Introduction

Ageing and obesity are two of the main public health challenges worldwide (Jura and Kozak, 2016). According to World Health Organization (WHO) reports, between 2020 and 2030, the number of individuals age 60 years and over will increase from 12% to 22% of the global population (Envejecimiento y salud), reflecting exponential growth in this age group. At the same time, obesity is one of the most relevant causes of global morbidity and mortality (Obesity). It is estimated that by 2030, approximately 1 in 5 women and 1 in 7 men will be living with obesity, surpassing 1 billion individuals worldwide (World obesity atlas 2022 | World obesity federation). The World Obesity Federation projects a 115% increase in adults with obesity between 2010 and 2030 (Marzuca-Nassr et al., 2023; Artigas-Arias et al., 2024).

Ageing is associated with structural and functional changes in various body systems, including the musculoskeletal system (Bouredji et al., 2022). A loss of approximately 10% of muscle mass has been described by the age of 50 years (Lexell et al., 1988), with an annual decline of 0.7%-0.8% after the age of 70 years (Koster et al., 2011). However, changes in skeletal muscle are not limited to muscle mass; they also involve muscle quality (Cruz-Jentoft et al., 2019). The concept of muscle quality integrates both the morphological and functional characteristics of skeletal muscle (Andersen, 2003; Larsson et al., 2019; Mahdy, 2019). These changes make muscle quality a strong predictor of functional independence and mortality in older adults (Linge et al., 2021).

From a morphological perspective, muscle quality considers parameters such as composition (intramuscular adipose tissue infiltration, [IMAT]) and muscle architecture (thickness, pennation angle, and fascicle length) (Fragala et al., 2015; Heymsfield et al., 2015). Functionally, it incorporates measurements of strength, power, and the strength to muscle mass ratio (de Lucena Alves et al., 2023). These parameters show negative alterations during ageing, which extrinsic factors like obesity may accelerate (Koliaki et al., 2019). Older adults with obesity exhibit lower muscle quality compared with their normal-weight counterparts, as well as a negative correlation between IMAT and muscle thickness (r = −0.395, p < 0.001) (Giuliani et al., 2020; Pereira et al., 2021). Moreover, it has been reported that older women experience a more pronounced deterioration in muscle quality than men during ageing (Ichinose et al., 2024). Regarding muscle distribution, lower-limb muscles appear to be the most susceptible to ageing, particularly the quadriceps (Ikezoe, 2020) muscle and its components – the rectus femoris and vastus intermedius – which experience greater losses in muscle quantity and quality (de Lucena Alves et al., 2023). Consequently, the quadriceps is the most frequently studied muscle in research on muscle mass and muscle quality.

Maintaining an adequate body composition, particularly in terms of muscle mass, can mitigate the adverse effects of ageing and obesity (Liu et al., 2023). In this context, resistance training (RT) has proven to be an effective strategy for increasing skeletal muscle mass, strength, and physical performance in older adults (Grgic et al., 2020). However, most studies have been conducted in older individuals without obesity, and evidence regarding the effects of RT in older adults with different body compositions remains limited. While obesity is associated with increased adiposity, chronic low-grade inflammation, insulin resistance, and IMAT – factors that may impair the anabolic and adaptive capacity of skeletal muscle in older adults with this condition (Mengeste et al., 2021; Park and Choi, 2023), the evidence suggests that RT is a promising therapy for reducing IMAT and improving mitochondrial dysfunction in obesity-induced muscle deterioration (Mengeste et al., 2021; Axelrod et al., 2023).

The available evidence on the effects of RT on muscle quality parameters in older women with different body compositions is still limited and heterogeneous. Some studies have reported smaller increases in muscle mass and thickness in older adults with obesity compared with older women with normal weight (Pina et al., 2018), suggesting that nutritional status may modulate training-induced adaptations in muscle mass (Tibana et al., 2017; Pina et al., 2018). In contrast, other studies have described similar responses between groups in terms of fat-free mass following an RT programme (Tibana et al., 2017). From a functional perspective, comparable improvements in muscle strength and power have been observed between older adults with and without obesity; however, a lower gain in muscle strength has also been reported in participants with obesity compared with their non-obese counterparts (Junior et al., 2021).

Despite these advances, there is a paucity of studies addressing the effects of RT on morphological and functional parameters of muscle quality in older adults with different body composition profiles (Radaelli et al., 2013; Seo et al., 2021). It is critical to generate evidence in this area to strengthen the scientific basis guiding exercise prescription in older women with and without obesity, considering not only muscle quantity but also muscle quality and physical performance. Therefore, the present study aimed to compare the effects of a 12-week RT programme on muscle quality and physical performance in women aged 60–79 years with obesity versus age-matched normal-weight women. We hypothesised that a 12-week RT programme improves muscle quality and physical performance in both older woman with a normal weight or obesity. Additionally, we hypothesised that women with obesity have greater baseline deficiencies, which could lead to a different response compared to women with a normal weight after training.

## Materials and methods

### Participants and study design

**Figure 1:**
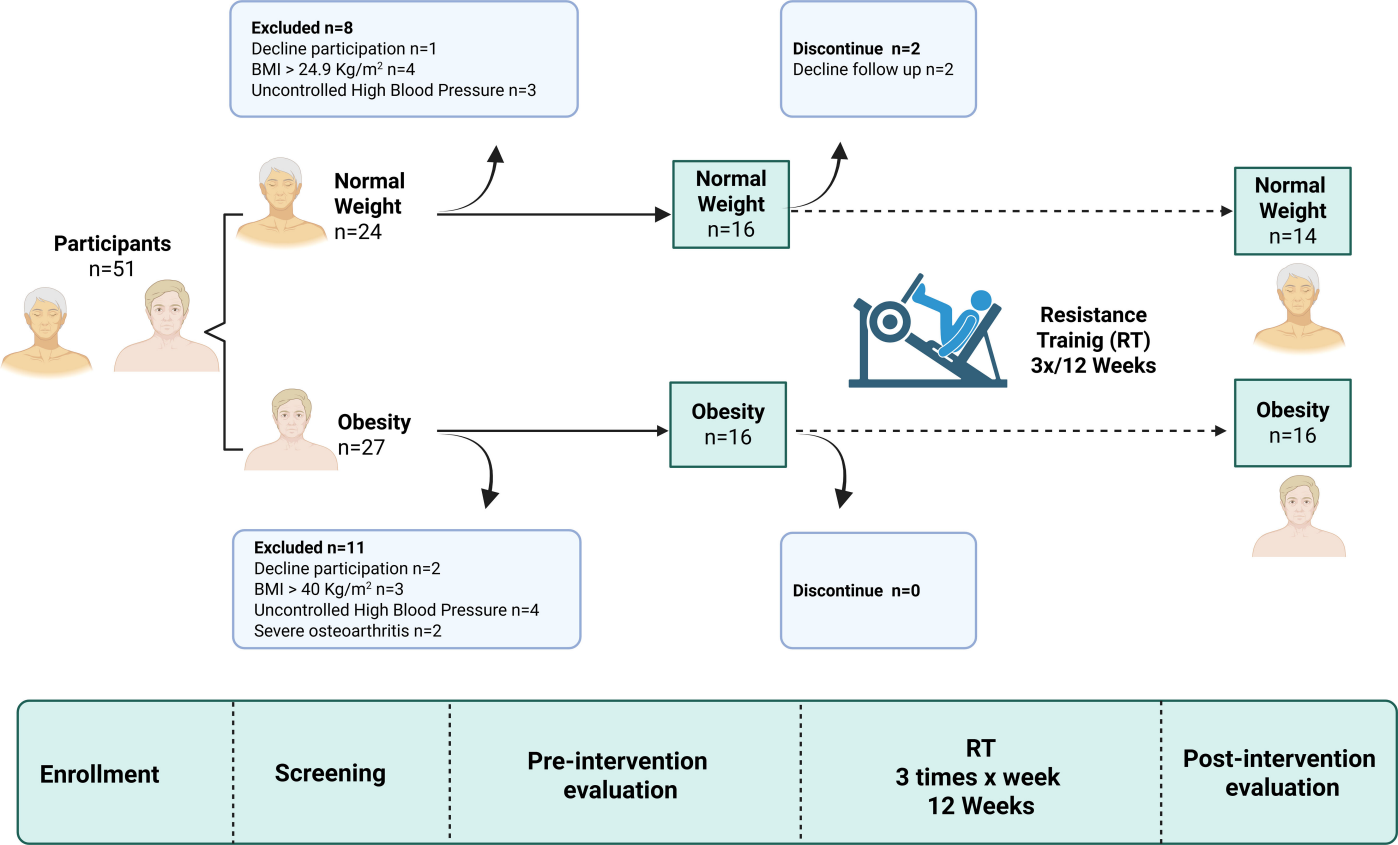
Flow diagram of study participants. Flow Chart. Fifty-one older women participants was enrollment to the study, from which 32 were included and 30 of them completed RT program, thus the final number of participants included were 14 older women of Normal Weight, and 16 older women with obesity. RT was performed 3 times a week for 12 weeks training from 60 to 80% of 1RM.

This prospective pre–post intervention study with parallel, naturally defined groups included 30 older women who completed the study and were allocated into two groups according to body composition: normal weight (NW, 66 ± 4.31 years; body mass index [BMI] = 22.60 ± 1.36 kg/m²; body fat % = 33.11% ± 3.61; n = 14) and obesity (OB, 68 ± 4.21 years; BMI = 33.01 ± 2.62 kg/m²; body fat % = 42.23% ± 2.98%; n = 16). There was no non-intervention control group (**Figure 1**). The sample size was estimated for two independent groups using G*Power 3.1.9.7, considering an effect size of 0.29, a significance level of α = 0.05, and a statistical power of 0.95. For this calculation, changes in quadriceps muscle thickness observed in previous studies by our research team in postmenopausal women undergoing the same 12-week training protocol and evaluated with the same ultrasound device were used as a reference (Artigas-Arias et al., 2024). Participants were recruited through social media, public announcements in the city of Temuco, and notice boards within the Universidad de La Frontera (Chile). The protocol was approved by the Scientific Ethics Committee of the Universidad de La Frontera, Temuco, Chile (code n°03/24), conducted in accordance with the principles of the Declaration of Helsinki, and registered in ClinicalTrials.gov (identifier: NCT06367296). All participants signed an informed consent form prior to the start of the study.

One week before the study, participants completed a general health medical questionnaire to confirm eligibility. The inclusion criteria were women aged 60–79 years, with a BMI of 18.5–24.9 kg/m² for the NW group and 30–39.9 kg/m² for the OB group. This classification was subsequently complemented by the assessment of additional parameters, including body fat percentage, waist circumference, and the waist-to-hip ratio, in order to achieve a more comprehensive characterisation of the obesity status. The exclusion criteria included regular RT within the past 6 months, medical conditions incompatible with physical training, comorbidities affecting mobility or preventing safe performance of RT, and the use of nutritional supplements (leucine, glutamine, casein, whey protein, or creatine).

The participants completed a supervised 12-week RT programme, consisting of whole-body exercises three times per week. Before and after the intervention, morphological parameters of the quadriceps muscle were assessed using muscle ultrasound, including muscle thickness as the primary study outcome and echointensity. To ensure measurement consistency, intra-rater reliability was assessed using the intraclass correlation coefficient (ICC). Total quadriceps muscle thickness showed an ICC of 0.956 (95% confidence interval [CI]: 0.956–0.988), and echointensity showed an ICC of 0.957 (95% CI: 0.922–0.978). The reported values correspond to the mean of three measurements performed by the same evaluator. These results confirm the high reliability of the measurements used in the study. Absolute and relative muscle strength (adjusted for total body mass), as well as lower-limb power, were measured as functional parameters of muscle quality. Body composition (muscle mass and total and segmental fat mass) was determined using whole-body bioelectrical impedance analysis (BIA). Physical performance was evaluated using the following tests: single-leg stance, Timed Up and Go (TUG), Five Times Sit-To-Stand (5-STS), and gait speed.

### Muscle mass and body composition

Absolute (kg) and relative (%) muscle mass, as well as body fat mass, were measured using BIA (Tanita MC-980U PLUS, Japan), employing the standard mode and accounting for each participant’s sex, age, and height. The participants were evaluated in a fasting state, ensuring that they had not engaged in intense physical activity during the previous 48 hours. Assessments were conducted following the manufacturer’s guidelines, with the participants standing barefoot, wearing light clothing, and without any metal object. Body weight and height were also assessed, and BMI was calculated according to previously reported procedures (Marzuca-Nassr et al., 2023; Artigas-Arias et al., 2024). Finally, hip and waist circumferences and the waist-to-hip ratio were measured using a Cescorf^®^ metallic tape measure calibrated in centimetres (CESCORF Equipamentos, Brazil).

### Morphological parameters of muscle quality

Ultrasound images were obtained using the LOGIQ™ F8 ultrasound system (GE Healthcare, USA) equipped with a 10-MHz linear transducer. Measurements of the vastus intermedius and rectus femoris were taken on the anterior thigh, with the participants in the supine position on an examination table and their knees fully extended. The assessment was conducted by an evaluator experienced in measuring muscle thickness using clinical ultrasonography, who was blinded to the group to which the participant was assigned. The transducer was placed transversely at the midpoint between the anterior superior iliac spine and the superior border of the patella of the dominant lower limb (Nijholt et al., 2020). The preset mode for skeletal muscle was used without modifying the frequency, gain, or focus parameters. To avoid tissue compression, the transducer was positioned with minimal pressure, using a layer of conductive gel (Artigas-Arias et al., 2024).

For each assessment, three consecutive images were acquired, and their mean was used. Muscle thickness was measured directly on the ultrasound device, and values were recorded in centimetres (Narici et al., 2021). Echointensity was subsequently analysed using the ImageJ software (National Institutes of Health, USA). For each image, a region of interest (ROI) of the rectus femoris muscle was selected using the polygon selection tool, avoiding the inclusion of bone tissue or fascial structures. The histogram function was used to obtain echointensity values, expressed in arbitrary units (AU) within a range from 0 (black) to 255 (white) (Bali et al., 2020). The mean value of the three images was then adjusted for subcutaneous tissue thickness (Young et al., 2015).

### Muscle strength

Maximal strength was assessed using the one-repetition maximum (1RM). Initially, 1RM was indirectly estimated during a familiarisation session through submaximal repetitions. Subsequently, in a separate session, 1RM was determined for each of the exercises included in the RT programme – leg extension, leg curl, and leg press (lower limbs), as well as chest press and elbow extension (upper limbs) – using the same machines employed during the training period. Additionally, handgrip strength was evaluated using a portable electronic Jamar dynamometer (Plus+ model, Patterson Medical, USA), as previously reported (Marzuca-Nassr et al., 2023). All maximal strength values were adjusted for each participant’s total body mass.

### Muscle power and muscle quality index

Muscle power, considered a functional parameter of muscle quality, was assessed using four muscle quality indexes (MQI): (1) STS mean power, (2) relative STS mean power, as proposed by Alcázar et al (Alcazar et al., 2018), (3) MQI1 proposed by Takai et al (Takai et al., 2009), and (4) MQI2 proposed by Brown et al (Brown et al., 2016). Each formula quantifies muscle power in watts (W) during the 5-STS. The formulas incorporate anthropometric and performance parameters, including leg length (m), chair height used in the 5-STS (0.45 m), body mass (kg), height (m), gravitational acceleration (9.81 m/s²), and the time recorded to complete the 5-STS (s).


$$\begin{array}{l} 1.\text{ STS mean power}\\ =\text{ Body mass x }0.9\text{ x g x }(\text{Height x }0.5\\ -\text{ Chair height})\;/\;(\text{Five STS time x }0.1) \end{array}$$

$$\begin{array}{l} 2.\text{ Relative STS mean power}\\ =\;0.9\text{ x g x }(\text{Height x }0.5\;-\text{ Chair height})\;/\;(\text{Five STS time x }0.1) \end{array}$$

3. MQI1= (Leg length x 0.45) x Body mass x g x 10/ Five STS time

4. MQI2= (Leg length − 0.5) x Body mass x g x 5 / Five STS time

Additionally, an MQI of muscle quality previously employed in the literature (de Lucena Alves et al., 2023) was considered. It was calculated by dividing strength – determined as the 1RM in the leg extension exercise (kg) – by the lower-limb muscle mass obtained through segmental BIA (Tanita MC-980U PLUS, Japan):


1RM leg extension÷lower limbs muscle mass

### Physical performance

Physical performance was assessed using the single-leg stance test, the Timed Up and Go (TUG) test, the 5-Repetition Sit-to-Stand (5-STS) test, and gait speed, as part of the Short Physical Performance Battery (SPPB) (Guralnik et al., 1994).

### Standardisation of dietary intake and physical activity

The participants were instructed to maintain their habitual dietary and physical activity patterns throughout the study. Dietary intake was assessed using a food frequency questionnaire and a 24-hour dietary recall during weeks – 1 and 11, aimed at estimating the average energy and macronutrient intake of the participants. From both sources, the average daily portions of different food groups were calculated, along with energy intake (kilocalories, [kcal]) and macronutrients (proteins, lipids, and carbohydrates), using the Food Composition Tables from the Institute of Nutrition and Food Technology (INTA), University of Chile, and the Atlas of Foods and Typical Preparations from the National Food Consumption Survey (ENCA; MINSAL, 2010). The requirements ware estimated through the direct method (Di Girolami, 2008). Intake values are expressed as daily averages in kcal and grams of proteins, lipids, and carbohydrates.

Physical activity was assessed using the International Physical Activity Questionnaire-Short Form (IPAQ) and the number of steps recorded over three days (OMRON pedometer, Japan). These control variables were measured during weeks – 1 and 11 to ensure consistency and reliability in assessing dietary intake and physical activity levels throughout the intervention.

### Resistance exercise training

Participants in both groups performed the same whole-body RT programme three times per week for 12 weeks (36 sessions in total). Each training session included three phases: warm-up, the main phase, and cool-down (Marzuca-Nassr et al., 2023; Artigas-Arias et al., 2024). The warm-up consisted of 5 minutes of aerobic exercise on a stationary bike, followed by general upper-limb mobility exercises. The main phase included machine-based exercises for the lower and upper body. Five sets of 10 repetitions were performed on lower-limb machines (leg press, leg extension, and leg curl; Fit Tech, USA) and three sets of 10 repetitions on upper-limb machines (chest press and elbow extension; Fit Tech, Portugal). The cool-down phase consisted of 5 minutes of general muscle stretching aimed at facilitating recovery.

During the first six weeks, the training load was progressively increased from 60% to 80% of the 1RM, maintaining 10 repetitions per set. At the end of this period, a new 1RM assessment was conducted to adjust training loads (60%–80% of 1RM) for the subsequent six weeks(Artigas-Arias et al., 2024). To be included in the protocol analyses, the participants were required to complete at least 80% of the training sessions, that is, a minimum of 29 of the 36 scheduled sessions.

### Statistical analysis

The data were analysed using the SPSS Statistics version 21.0 (IBM Corp., Armonk, NY, USA), while figures were generated using GraphPad Prism 8.2 (GraphPad Software, San Diego, CA, USA). The results are presented as the mean ± standard deviation (SD). Comparisons of baseline characteristics and percentage changes for each variable between groups were analysed using an independent-samples t-test.

To evaluate pre- and post-intervention changes, a repeated-measures ANOVA was applied, considering time (PRE vs. POST) as the within-subject factor and group (NW vs. OB) as the between-subject factor. Prior to the repeated-measures ANOVA, the assumptions of normality, homogeneity of variances, and sphericity were assessed using the Shapiro–Wilk test, Levene’s test, and Mauchly’s test of sphericity, respectively. In the case of a significant interaction, paired t-tests were conducted to analyse within-group time effects, and independent t-tests were used to evaluate group differences at pre- and post- intervention.

The baseline effect size between the groups was estimated using Cohen’s d, which was interpreted as follows <0.2, no effect; 0.2–0.49, a small effect; 0.5–0.79, a medium effect; and ≥0.8, a large effect (Cohen, 2013). Partial eta squared (η²) was also used to estimate effect sizes from the ANOVA calculations, with 0.01 indicating a small effect, 0.06 a medium effect, and 0.134 or higher a large effect. Statistical significance was set at p<0.05.

## Results

### Baseline characteristics

The baseline characteristics of the participants are shown in **Table 1**. There were no differences between the NW and OB groups in age, height, heart rate, or blood pressure (p > 0.05). However, as expected, both groups showed significant differences in body composition and muscle quality variables (p < 0.05). Two participants withdrew during the study, as detailed in the flow diagram (**Figure 1****).**

**Table 1:** Participants characteristics.

Characteristics	Older normal weight (n=14)	Older obesity (n=16)	p value
Age (years)	66 ± 4	68 ± 4	0.270
Weight (kg)	55.66 ± 4.49	80.98 ± 9.77	**0.000**
Height (m)	1.57 ± 0.07	1.56 ± 0.06	0.902
BMI (kg·m−2)	22.60 ± 1.36	33.01 ± 2.62	**0.000**
Waist circumference (cm)	81.54 ± 5.52	104.28 ± 7.06	**0.000**
Waist-to hip ratio	0.87 ± 0.06	0.92 ± 0.05	**0.017**
Muscle mass (kg)	35.39 ± 3.38	44.16 ± 4.57	**0.000**
% Muscle mass	63.76 ± 3.04	54.68 ± 2.82	**0.000**
% Fat mass	33.11 ± 3.61	42.23 ± 2.98	**0.000**
Visceral fat (kg)	7.57 ± 1.09	12.66 ± 1.45	**0.000**
Quadriceps thickness (cm)	3.01 ± 0.47	3.85 ± 0.62	**0.000**
% Quadriceps thickness	70.98 ± 3.77	64.48 ± 9.63	0.025
1RM leg extension (Kg)	49.07 ± 16.77	50.54 ± 12.25	0.785
Relative 1RM leg extension	0.88 ± 0.27	0.62 ± 0.14	**0.002**
HR (b·min−1)	68.71 ± 7.87	73.44 ± 8.76	0.134
SBP (mm Hg)	121.57 ± 12.00	126.94 ± 9.28	0.179
DPB (mm Hg)	72.86 ± 6.41	76.56 ± 8.49	0.193

n, number of participants; BMI, body mass index; HR, heart rate; SBP, systolic blood pressure; DBP, diastolic blood pressure. Relative, variable/ body weight. Values represent means ± SD. Bold values indicated different between NW and OB at the p < 0.05 level.

**Figure 2:**
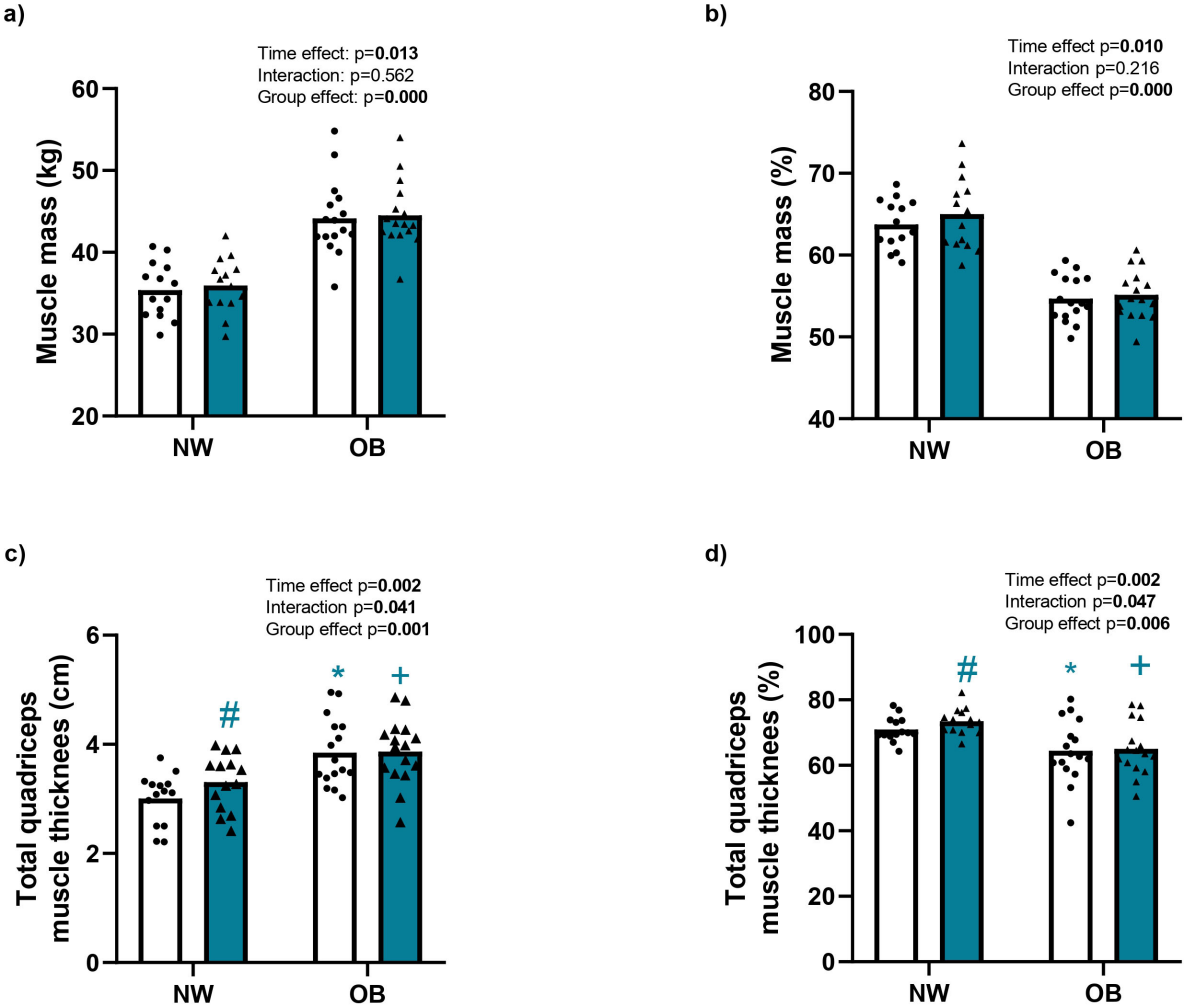
Effect of RT up muscle mass and thickness. NW, Normal Weight; OB, Obesity, **(a)** Muscle mass, **(b)** percentage of muscle mass, **(c)** thickness total quadriceps muscle and **(d)** percentage thickness total quadriceps muscle of the dominant leg before and after 12 weeks of resistance training; • Pre and ♦ Post. Data were analyzed using repeated-measure ANOVA (time x group); Bold values indicate difference at the p < 0.05; paired t-tests: # (p = 0.003) between pre vs post NW; independent t-tests: * (p < 0.017) between pre NW vs pre OB; independent t-tests: + (p < 0.011) between post NW vs post OB.

**Figure 3:**
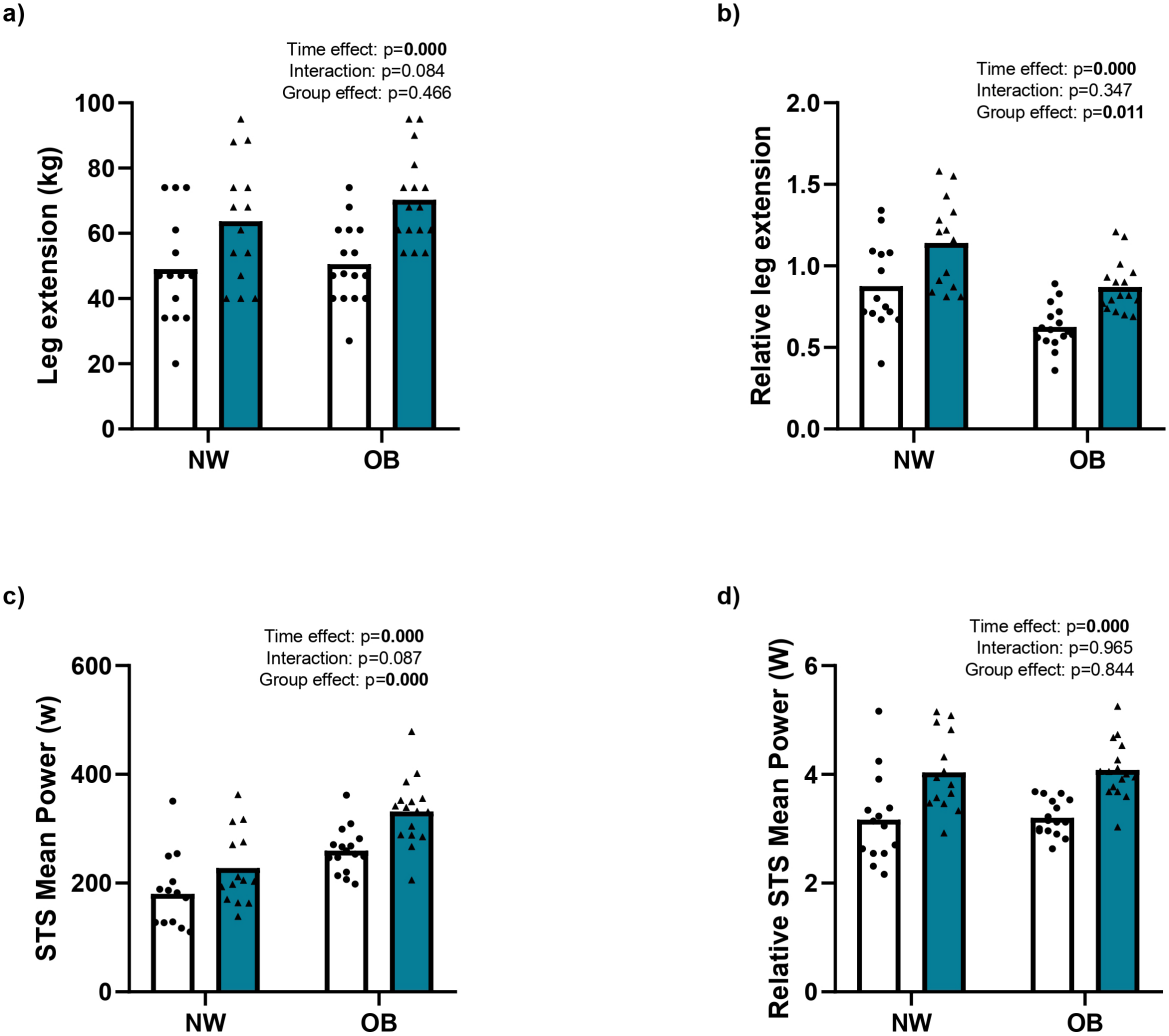
Effect of RT up strength and power. NW, Normal Weight, OB, Obesity, **(a)** Leg extension (kg), **(b)** percentage of leg extension, **(c)** STS mean power and **(d)** relative STS mean power before and after 12 weeks of resistance training, • Pre and♦Post. Data were analyzed using repeated-measure ANOVA (time x group); Bold values indicate difference at the p < 0.05.

In absolute terms, the OB group presented higher muscle mass, thickness, strength, and power compared with the NW group (group factor, p < 0.009; η² > 0.221), except for the following variables: 1RM leg extension (group factor, p = 0.466; η² = 0.019), 1RM leg press (group factor, p = 0.251; η² = 0.041), 1RM leg curl (group factor, p = 0.062; η² = 0.119), and relative STS power (group factor, p = 0.844; η² = 0.000). However, when adjusted for body weight or expressed as percentages, the muscle quantity and quality variables were higher in the NW group compared with the OB group (group factor, p < 0.035; η² > 0.149) (**Figures 2**, **3**; **Tables 2**, **3****).**

**Table 2:** Effect of RT on morphological of muscle quality.

Measurements	Older normal weight (n=14)			Older obesity (n=16)			Whithin-subjects effects		Between-subjets effects
	Pre	Post	%Pre-post	Pre	Post	%Pre-post	Time	Time * group	Group
Muscle thickness and echointensity									
RF (cm)	1.66 ± 0.36	1.88 ± 0.30**#**	16.23 ± 21.44	2.05 ± 0.27*	2.06 ± 0.25	0.92 ± 6.54	**0.004**	**0.009**	**0.009**
RF (%)	39.01 ± 5.82	41.76 ± 4.11	8.43 ± 13.31	34.46 ± 4.92	34.95 ± 4.70	1.70 ± 5.65	**0.011**	0.069	**0.002**
VI (cm)	1.21 ± 0.30	1.29 ± 0.31	7.93 ± 17.24	1.63 ± 0.39	1.69 ± 0.44	4.79 ± 19.35	0.154	0.836	**0.003**
VI (%)	28.46 ± 5.25	28.51 ± 5.03	1.41 ± 15.17	27.24 ± 5.77	28.,16 ± 5.47	4.79 ± 14.40	0.513	0.558	0.670
RF (A.U.)	114.93 ± 9.82	114.49 ± 10.87	-0.29 ± 5.87	100.00 ± 13.35	101.77 ± 10.74	2.50 ± 9.41	0.661	0.467	**0.001**

RT, Resistance training; PRE, before RT; POST, after RT; n, number of patients; RF, Rectus femoris; VI, Vast intermedius; **%**, Percentage of muscle relative to total thigh thickness, determined by ultrasound. Values represent means ± SD; Bold values indicate difference at the p < 0.05; paired t-tests: **#** (p < 0.003) between PRE vs POST; independent t-tests: * (p < 0.017) between PRE NW vs PRE OB.

**Table 3:** Effect of RT on functional parameters of muscle quality.

Measurements	Older normal weight (n=14)			Older obesity (n=16)			Whithin-subjects effects		Between-subjets effects
	Pre	Post	%Pre-post	Pre	Post	%Pre-post	Time	Time * group	Group
Strength (1RM)									
Leg press (kg)	56.29 ± 20.10	78.29 ± 20.62	47.26 ± 37.30	60.90 + 16.01	89.39 ± 19.69	50.02 ± 25.05	**0.000**	0.116	0.251
Relative leg press	1 ± 3.31	1.42 ± 0.34	48.61 ± 39.72	0.77 + 0.23	1.11 ± 0.26	49.86 ± 25.15	**0.000**	0.347	**0.011**
Curl flexion (kg)	39.64 ± 12.58	47.86 ± 12.97	24.96 ± 25.05	44.69 + 9.74	57.13 ± 7.37	31.63 ± 23.63	**0.000**	0.136	0.062
Relative curl flexion	0.71 ± 0.20	0.86 ± 0.19	22.77 ± 25.92	0.56 + 1.28	0.71 ± 0.08	31.36 ± 22.32	**0.000**	0.945	**0.008**
Chest press (kg)	39.64 ± 10.09	50.71 ± 10.16	33.88 ± 41.91	50.00 ± 9.31	64.06 ± 11.14	29.35 ± 15.27	**0.000**	0.252	**0.002**
Relative chest press	0.71 ± 0.16	0.92 ± 0.17	34.98 ± 43.98	0.62 ± 0.12	0.79 ± 0.12	29.11 ± 14.21	**0.000**	0.413	**0.028**
Elbow extension (kg)	20.00 ± 5.55	30.00 ± 6.50	57.62 ± 45.71	26.88 ± 36.88	36.88 ± 4.03	43.10 ± 34.57	**0.000**	1.000	**0.000**
Relative elbow extension	0.36 ± 0.08	0.54 ± 0.10	58.67 ± 46.24	0.33 ± 0.08	0.46 ± 0.06	43.06 ± 35.36	**0.000**	0.078	**0.035**
Muscle quality indexes									
MQI (W)	223.00 ± 72.36	282.27 ± 70.24	30.00 ± 21.54	319.79 ± 36.12	409.83 ± 69.86	28.40 ± 17.97	**0.000**	0.092	**0.000**
MQI2 (W)	100.62 ± 39.64	127.21 ±40.88	29.99 ± 21.54	138.67 ± 20.68	178.79 ± 40.65	28.40 ± 17.97	**0.000**	0.116	**0.001**
1RM leg extension/lower limbs muscle mass	4.37 ± 1.27	5.61 ± 1.35	31.72 ± 22.59	3.65 ± 0.72	5.01 ± 0.90	40.05 ± 24. 31	**0.000**	0.589	0.092

RT, Resistance training; PRE, before RT; POST, after RT; n, number of patients; MQI, Muscle quality index. Values represent means ± SD; Bold values indicate difference at the p < 0.05.

### Effects of 12 weeks of resistance training

#### Muscle mass and body composition

After 12 weeks of RT, there was a significant increase in absolute muscle mass (time effect, p = 0.013; η² = 0.201), increasing from 35.39 ± 3.38 to 35.96 ± 3.34 kg (a 1.67% ± 2.17% increase) in the NW group and from 44.16 ± 4.57 to 44.53 ± 4.04 kg (a 0.80% ± 2.55% increase) in the OB group. Similarly, there was a significant increase in muscle mass percentage (time effect, p = 0.010; η² = 0.214), rising from 63.76% ± 3.04% to 65.01% ± 4.46% (a 1.89% ± 2.95% increase) and from 54.58% ± 2.82% to 55.14% ± 2.98% (a 0.87% ± 2.69% increase) in the NW and OB groups, respectively. The time x group interaction was not significant for these variables (interaction, p > 0.216; η² < 0.054) (**Figures 2a, b**).

The effects of 12 weeks of RT on the remaining body composition variables are presented in **Table 4**. There was a significant decrease in body fat percentage (time effect, p = 0.012; η² = 0.206), waist circumference (time effect, p = 0.016; η² = 0.190), the waist-to-hip ratio (time effect, p = 0.028; η² = 0.161), and visceral fat (time effect, p = 0.007; η² = 0.231). Regarding the time × group interaction, there was a significant effect for body fat percentage (p = 0.040; η² = 0.142), with a significant reduction within the NW group (p = 0.010), and no significant change in the OB group (p = 0.671). Body weight and BMI showed no significant changes (p > 0.804; η² < 0.002).

**Table 4:** Effect of RT on body composition.

Measurements	Older normal weight (n=14)			Older obesity (n=16)			Whithin-subjects effects		Between-subjets effects
	Pre	Post	%Pre-post	Pre	Post	%Pre-post	Time	Time * group	Group
Weight (kg)	55.66± 4.49	55.44 ± 7.55	-0.58 ± 2.75	80.98 ± 9.77	81.07 ± 9.74	0.12 ± 1.46	0.804	0.555	**0.000**
BMI (Kg/m^2^)	22.60 ± 1.36	22.62 ± 1.60	0.09 ± 2.75	33.01 ± 2.62	33.03 ± 2.74	0.04 + 1.29	0.817	0.969	**0.000**
% Fat mass	33.11 ± 3.61	31.87 ± 4.06**#**	-4.62 ± 9.90	42.23 ± 2.98*	42.09 ± 3.25**^♦^**	0.06 ± 3.46	**0.012**	**0.040**	**0.000**
Waist Circumference (cm)	81.54 ± 5.52	79.87 ± 5.59	-1.96 ± 4.69	104.28 ± 7.06	102.67 ± 7.30	-1.52 ± 3.05	**0.016**	0.964	**0.000**
Visceral fat (kg)	7.57 ± 1.09	7.43 ± 1.28	-1.88 ± 9.95	12.63 ± 1.45	11.94 ± 1.24	-5.17 ± 5.77	**0.007**	0.068	**0.000**
Waist-to hip ratio	0.87 ± 0.06	0.83 ± 0.03	-4.21 ± 6.76	0.92 ± 0.05	0.90 ± 0.09	-2.1 ± 7.63	**0.028**	0.404	**0.006**

PRE, before RT; POST, after RT; n, number of participants; Values represent means ± SD; Bold values indicate difference at the p < 0.05; paired t-tests: **#** (p = 0.001) between PRE vs POST; independent t-tests: * (p < 0.01) between PRE NW vs PRE OB; independent t-tests: **♦**(p = 0.000) between POST NW vs POST OB.

#### Morphological parameters of muscle quality

The effects of 12 weeks of RT on muscle thickness, expressed in absolute terms (cm) and as a percentage (%), are presented in **Figures 2c, d**; **Table 2**. There was a significant increase in quadriceps muscle thickness, increased from 3.01 ± 0.47 to 3.31 ± 0.51 cm (a 10.56% ± 10.13% increase) in the NW group and from 3.85 ± 0.62 to 3.87 ± 0.60 cm (a 1.03% ± 10.68% increase) in the OB group (time effect, p = 0.002; η² = 0.173; **Figure 2c**). When expressed as a percentage, quadriceps thickness increased from 70.98% ± 3.77% to 73.49% ± 3.90% (a 3.59% ± 3.79% increase) in the NW group and from 64.48% ± 9.63 to 65.08% ± 8.14% (a 1.44% ± 5.15% increase) in the OB group (time effect, p < 0.002; η² = 0.290; **Figure 2d**). There was a significant time × group interaction for quadriceps thickness (absolute: p = 0.041; η² = 0.140; %: p = 0.047; η² = 0.133; **Figure 2c, d**). Specifically, quadriceps thickness (absolute and relative) differed between the groups both before and after 12 weeks of RT (p < 0.05). Only the NW group showed a significant increase after the intervention (p < 0.05).

The analysis of the rectus femoris muscle is shown in **Table 2**. There was a significant increase after 12 weeks of RT (absolute: time effect, p = 0.004; η² = 0.258; %: time effect, p = 0.011; η² = 0.209; **Table 2**). The time × group interaction was significant for absolute rectus femoris thickness (p = 0.009; η² = 0.221) but not for relative thickness (p = 0.069; η² = 0.133). Specifically, absolute rectus femoris thickness differed between the groups before the 12-week RT intervention (p < 0.05). Only the NW group showed a significant increase after the intervention (p < 0.05).

The analysis of IMAT, evaluated via echointensity, yielded no significant changes following the intervention (time effect, p = 0.661; η² = 0.007).

#### Muscle strength

After 12 weeks of whole-body RT, maximal strength (1RM) for leg extension increased significantly, from 49.07 ± 16.77 kg to 63.68 ± 18.80 kg (a 33.74% ± 23.91% increase) in the NW group, and from 50.54 ± 12.25 kg to 70.31 ± 13.95 kg (a 42.64% ± 25.23% increase) in the OB group (time effect, p = 0.000; η² = 0.835; **Figure 3a**). When 1RM was adjusted for body weight, there were similar percentage increases, rising from 1.0 ± 0.31 to 1.42 ± 0.34 (a 34.55% ± 24.18% increase) in the NW group and from 0.77 ± 0.23 to 1.11 ± 0.26 (a 42.29% ± 23.70% increase) in the OB group (time effect, p = 0.000; η² = 0.828; **Figure 3b**). The time × group interactions were not significant (p > 0.116; η² < 0.032; **Figures 3a, b**).

For the remaining 1RM evaluations (chest press, elbow extension, leg press, and leg curl), there were similar gains in absolute and relative strength after 12 weeks of RT in both groups (time effect, p < 0.001; η² > 0.667). The time × group interactions were not significant (p > 0.078; η² < 0.019; **Table 3**).

#### Muscle quality indexes

Muscle quality assessed using STS mean power, relative STS power, MQI1, and MQI2 increased significantly after 12 weeks of RT (time effect, p < 0.001; η² > 0.696; **Figures 3c, d**; **Table 3**). STS mean power increased from 180.56 ± 67.85 to 227.68 ± 68.27 W (a 30.00% ± 21.54% increase) in the NW group and from 259.45 ± 41.57 to 331.79 ± 61.97 W (a 28.40% ± 17.97% increase) in the OB group (**Figure 3c**). When adjusted for body weight, the relative STS power increased from 3.17 ± 0.82 to 4.04 ± 0.71 W (a 30.83% ± 21.63% increase) in the NW group and from 3.20 ± 0.33 to 4.08 ± 0.53 W (a 28.13% ± 16.94% increase) in the OB group (**Figure 3d**). The time × group interactions were not significant (p > 0.087; η² < 0.101; **Figures 3c, d**).

The MQI1 and MQI2 results are presented in **Table 3**. They showed significant improvements in muscle quality (time effect, p < 0.000; η² > 0.494) but without significant time × group interactions (p > 0.092; η² < 0.098). Finally, muscle quality expressed as the ratio of lower-limb strength to muscle mass also increased after 12 weeks of RT (time effect, p < 0.000; η² > 0.822).

### Physical performance

After 12 weeks of RT, tehre were significant improvements in the single-leg stance, TUG, and 5-STS tests and gait speed (time effect, p < 0.043; η² > 0.238). The time × group interactions were not significant (p > 0.304; η² < 0.070; **Table 5**).

**Table 5:** Effect of RT on physical performance.

Measurements	Older normal weight (n=14)			Older obesity (n=16)			Whithin-subjects effects		Between-subjets effects
	Pre	Post	%Pre-post	Pre	Post	%Pre-post	Time	Time * group	Group
Single-leg stance test (s)	39.42 ± 22.41	48.47 + 17.54	22.96 +21.73	31.03 ± 22.61	41.64 ± 20.37	34.19 ± 22.69	**0.000**	0.743	0.304
TUG (s)	7.11 ± 1.21	6.35 ± 0.96	-9.76 ± 11.76	7.34 ± 1.04	6.27 ± 0.49	-13.72 ± 7.85	**0.000**	0.370	0.803
Walk speed (m/s)	1.52 ± 0.26	1.54 ± 0.26	1.97 ± 10.40	1.45 ± 0.22	1.56 ± 0.18	9.02 ± 13.94	**0.043**	0.157	0.738
5-STS (s)	9.63 ± 1.55	7.39 ± 0.79	-21.74 ± 12.12	9.22 ± 1.55	7.26 ± 0.92	-20,60 ± 11,05	**0.000**	0.574	0.428

PRE, before RT; POST, after RT; n, number of participants; TUG, Time Up go; 5-STS, 5-Repetition Sit-to-Stand test; Values represent means ± SD; Bold values indicate difference at the p < 0.05.

### Dietary intake and physical activity levels

Data on physical activity and dietary intake are presented in **Tables 6**, **7**, respectively. There were no significant differences in the macronutrient composition of the diet before and after the intervention. Similarly, there were no significant changes after 12 weeks of RT in moderate physical activity levels (time effect, p = 0.585; η² = 0.010), sedentary behaviour (time effect, p = 0.320; η² = 0.035), or daily step count (time effect, p = 0.445; η² = 0.045). As expected, due to the RT intervention, vigorous physical activity levels increased after 12 weeks of RT (time effect, p=0.000; η²=0.383).

**Table 6:** Level of physical activity before and at the end (week 11) of resistance exercise training.

Measurements	Older normal weight (n=14)		Older obesity (n=16)		Whithin-subjects effects		Between-subjets effects
	Pre	Post	Pre	Post	Time	Time * group	Group
Moderate physical activity (Met/min/sem)	592.86 ± 578.36	528.57 ± 563.11	1140.00 ± 1730.11	1481.25 ± 1630.97	0.585	0.426	0.071
Vigorous physical activiy (Met/min/sem)	394.29 ± 561.74	942.86 ± 705.79	165.00 ± 358.33	1030.00 ± 602.66	**0.000**	0.359	0.555
Seated (min)	450.71 ± 149.48	417.86 ± 195.85	473.13 ± 162.06	421.25 ± 155.37	0.320	0.822	0.772
Steps (n°)	5019.21 ± 2187.55	5779.79 ± 1346.31	3647.38 ± 1346.31	3694.39 ± 881.69	0.445	0.499	**0.006**

Data are means ± SD. Data were analyzed using repeated measures ANOVA (time x group). Bold values indicate difference at the p < 0.05.

**Table 7:** Dietary intake before and at the end (week 11) of resistance exercise training.

Measurements	Older normal weight (n=14)		Older obesity (n=16)		Whithin-subjects effects		Between-subjets effects
	Pre	Post	Pre	Post	Time	Time * group	Group
Protein intake (g)	66.93 ± 13.61	62.36 ± 16.68	68.47 ± 11.78	63.80 ± 13.71	0.070	0.985	0.748
Lipids intake (g)	95.36 ± 25.66	99.36 ± 29.43	95.60 ± 26.67	96.60 ± 27.46	0.531	0.706	0.894
Carbohydrates intake (g)	181.29 ± 37.46	188.07 ± 50.23	229.47 ± 56.97	226.47 ± 59.75	0.783	0.478	**0.024**
Total energy intake (Kcal)	1849.36 ± 320.65	1893.64 ± 412. 83	2050.67 ± 411.01	2029.73 ± 372.19	0.844	0.583	0.202
Estimation of energy requirements (Kcal)	2226.74 ± 183.20	2212.25 ± 209.23	1880.74 ± 157.18	1881.70 ± 153.52	0.321	0.216	**0.000**
Aceptable average Weight (kg)	63.62 ± 5.23	63.21 ± 5.98	62.67 ± 5.24	62.72 ± 5.12	0.341	0.218	0.722
% adequacy of calorie intake	83.08 ± 12.55	85.42 ± 16.49	109.21 ± 20.32	107.89 ± 17.42	0.864	0.540	**0.000**
Estimation of protein requirements (g)	76.34 ± 6.28	75.85 ± 7.17	75.20 ± 6.29	75.27 ± 6.14	0.341	0.218	0.722
% Estimation of protein requirements	87.99 ± 19.64	91.33 ± 16.60	91.33 ± 16.60	84.96 ± 18.45	0.077	0.997	0.589
Estimation of lipids requirements (g)	49.48 ± 4.07	49.16 ± 4.65	41.78 ± 3.49	41.82 ± 3.41	0.321	0.216	**0.000**
% Estimation of lipids requirements	191.42 ± 43.05	199.77 ± 48.81	229.12 ± 62.60	231.42 ± 65.81	0.574	0.749	0.074
Estimation of carbohydrate requirements (g)	334.01 ± 27.48	331.84 ± 31.38	282.01 ± 23.58	282.25 ± 23.03	0.321	0.216	**0.000**
% Estimation of carbohydrate requirements	54.54 ± 11.49	57.11 ± 16.37	81.29 ± 18.55	80.02 ± 19.47	0.776	0.406	**0.000**

Dietary intake for day; Data are means ± SD. Data were analyzed using repeated measures ANOVA (time x group). Bold values indicate difference at the p < 0.05.

## Discussion

This study aimed to compare the effects of 12 weeks of RT on muscle quality and physical performance in older women (aged 60–79 years) with obesity versus age-matched normal-weight women. The findings demonstrate that a progressive, whole-body RT programme induces structural and functional adaptations in skeletal muscle. There were improvements in muscle mass, lower-limb strength and power, as well as physical performance based on the single-leg stance, TUG, gait speed, and 5-STS tests. The time × group interaction analysis showed that only the NW group experienced a significant increase in muscle thickness and a reduction in body fat percentage compared with the OB group. It should be noted that there were no significant changes after 12 weeks of RT in either dietary intake or physical activity levels.

Ageing is associated with significant changes in the musculoskeletal system (McCormick and Vasilaki, 2018). The progressive loss of skeletal muscle mass and strength, accompanied by reduced physical performance (Cruz-Jentoft et al., 2019), Is linked to a higher risk of falls, disability, and mortality (Papadopoulou, 2020). On the other hand, obesity is characterised by increased and ectopically redistributed adipose tissue towards organs such as the liver, pancreas, and skeletal muscle (Kuk et al., 2009). IMAT infiltration is associated with mitochondrial dysfunction, lipotoxicity (Sachs et al., 2019; Li et al., 2022), and altered secretion of pro- and anti-inflammatory cytokines (Costamagna et al., 2015). These changes directly affect musculoskeletal structure and function, making obesity an extrinsic factor that can accelerate the development of sarcopenia in older adults (Koliaki et al., 2019).

Our results confirm significant differences between the groups in total muscle mass as well as in muscle thickness, strength, and power. Older women with obesity exhibited higher absolute values for total muscle mass and quadriceps thickness, strength, and power. However, when these parameters were normalised to by body weight, the OB group showed lower relative muscle mass, thickness, strength, and power compared with the NW group (**Figures 2**, **3**; **Tables 2**, **3**). These findings align with previous reports indicating that absolute values may underestimate the real impact of obesity on muscle quality (Tomlinson et al., 2015) and the risk of mortality in this population (Kim et al., 2022).

Although RT is widely supported for older adults, many studies exclude individuals with obesity, limiting knowledge about muscle responses in this population. In this study, 12 weeks of RT resulted in a significant increases in muscle mass over time, with no evidence of a time x group interaction. However, this increase was quantitatively smaller in the participants with obesity, inducing gains of 1.89% and 0.87% in total muscle mass in the NW and OB groups, respectively (**Figure 2**). These gains are consistent with previous studies reporting a ~2% total lean mass increase in non-obese older adults after 12 weeks of RT (Marzuca-Nassr et al., 2023), confirming the effectiveness of progressive RT to improve musculoskeletal structure and function in older adults (Leenders et al., 2013; Churchward-Venne et al., 2015). Ultrasound analysis of muscle thickness revealed a significant increase after 12 weeks of RT. Nevertheless, there was a significant time × group interaction for total quadriceps thickness (absolute and relative values; **Figures 2c, d**) and for the rectus femoris (absolute values; **Table 2**), indicating that only the NW group showed significant increases in these variables.

A possible explanation for these findings is the presence of obesity-related anabolic resistance (Nilsson et al., 2024). Factors such as chronic low-grade inflammation, lipid accumulation within muscle, and altered metabolic signalling have been proposed to impair muscle protein synthesis in response to RT (Churchward-Venne et al., 2015; Zhang et al., 2021; Liberale et al., 2022). However, given that we did not assess these mechanisms directly, and considering the variability in our data, this explanation should be viewed as a potential contributing factor rather than a definitive one. More broadly, our findings are in line with previous research showing that RT can lead to meaningful improvements in muscle strength and function even when changes in muscle size are limited (McGregor et al., 2014).

In this same context, the heterogeneity observed in individual training responses is consistent with studies highlighting inter-individual variability in physiological adaptations, influenced by personal characteristics, the training protocol, and the variable analysed (Bouchard and Rankinen, 2001; Bonafiglia et al., 2016; Formighieri et al., 2022). Nonetheless, all older adults benefit from RT, particularly with longer interventions, reinforcing the need to promote this training modality without restrictions in the older population (Churchward-Venne et al., 2015). This type of variability is commonly reported in RT studies in older adults and likely reflects differences in individual responsiveness rather than clear physiological divergence between groups (Ahtiainen et al., 2016).

The musculoskeletal system does not just undergo quantitative changes in muscle mass (Cruz-Jentoft et al., 2019). Strong evidence demonstrates that muscle strength and power are more closely related to mobility, functionality, and mortality in older adults than muscle mass alone (Fragala et al., 2015). In this context, “muscle quality” integrates morphological parameters (composition and architecture) with functional parameters (strength, power, and MQI) (Fragala et al., 2015).This study shows that 12 weeks of RT modified the morphological parameters of muscle quality, such as muscle thickness, but not echointensity (Vincenty et al., 2025). There were no significant changes in echointensity after 12 weeks of RT. These findings are consistent with previous reports showing that older women with a normal weight (70.3 ± 5.38 years; BMI = 22.9 ± 2.02 kg·m^-2^) undergoing 16 weeks of elastic-band RT did not show reductions in IMAT (Seo et al., 2021). Conversely, Radaelli et al., (2013). observed decreased IMAT in the quadriceps via computed tomography after RT in older women with a normal weight. Similarly, Flor-Rufino et al., (2023). demonstrated that 6 months of high-intensity RT reduced IMAT (based on magnetic resonance imaging) in older women with sarcopenic, and Ikezoe et al., (2020). observed an 8.05%–16.3% reduction in quadriceps muscle echointensity following an 8-week RT programme as assessed by ultrasound in young men. To our knowledge, this is the first study that has evaluated echointensity via ultrasound after 12 weeks of RT in older women with a normal weight and obesity. Hence, further research is needed to determine changes in echointensity among different populations and with different body compositions.

Regarding functional parameters, our results confirmed the widely reported beneficial effects of RT on muscle strength and power, MQI, and physical performance (Churchward-Venne et al., 2015; Guizelini et al., 2018; Fiogbé et al., 2019; Marzuca-Nassr et al., 2023), although the differences between the NW and OB groups were not significant. These findings confirms that MQI (Fragala et al., 2014) are key indicators of functionality in older women with obesity and normal weight independent of the percentage of muscle mass gain. Finally, the results showed that after 12-weeks of RT, despite no change in muscle thickness, improvements in strength, muscle power, and functional performance were still comparable between the NW and OB groups. This reinforces the idea that functional adaptations may be more reliable indicators of training effectiveness than changes in muscle thickness alone. Collectively, these findings underscore the clinical relevance of RT in older women with obesity.

In conclusion, prolonged RT improves muscle strength and power, MQI, and physical performance in older women. Although adaptations such as increases in muscle thickness and reductions in body fat percentage were only significant in the NW group, these between-group differences should be interpreted with caution, particularly given the relatively small sample size and the variability in individual responses. However, 12 weeks of RT are effective in improving functional parameters of muscle quality and physical performance in older women with a normal weight and obesity. These findings support RT as an effective strategy to optimize musculoskeletal health in this population, including those with obesity. Future studies with larger samples and more detailed mechanistic assessments are needed to better understand the factors contributing to variability in hypertrophic responses in older women with obesity.

### Limitations

Although the sample size was sufficient for the primary outcome, the large number of variables analysed may have benefited from a larger number of participants. Each morphological and functional variable was analszed independently, and formal adjustments for multiple comparisons were not applied. Additionally, body composition was assessed using BIA, a rapid, non-invasive method validated in older adults, although it is not considered the gold standard. Finally, this study lacked mechanistic assessments that could help explain the observed differences in morphological responses between the groups.

## Data Availability

The raw data supporting the conclusions of this article will be made available by the authors, without undue reservation.
